# Deep-Sea Bioluminescence Blooms after Dense Water Formation at the Ocean Surface

**DOI:** 10.1371/journal.pone.0067523

**Published:** 2013-07-10

**Authors:** Christian Tamburini, Miquel Canals, Xavier Durrieu de Madron, Loïc Houpert, Dominique Lefèvre, Séverine Martini, Fabrizio D'Ortenzio, Anne Robert, Pierre Testor, Juan Antonio Aguilar, Imen Al Samarai, Arnaud Albert, Michel André, Marco Anghinolfi, Gisela Anton, Shebli Anvar, Miguel Ardid, Ana Carolina Assis Jesus, Tri L. Astraatmadja, Jean-Jacques Aubert, Bruny Baret, Stéphane Basa, Vincent Bertin, Simone Biagi, Armando Bigi, Ciro Bigongiari, Claudio Bogazzi, Manuel Bou-Cabo, Boutayeb Bouhou, Mieke C. Bouwhuis, Jurgen Brunner, José Busto, Francisco Camarena, Antonio Capone, Christina Cârloganu, Giada Carminati, John Carr, Stefano Cecchini, Ziad Charif, Philippe Charvis, Tommaso Chiarusi, Marco Circella, Rosa Coniglione, Heide Costantini, Paschal Coyle, Christian Curtil, Patrick Decowski, Ivan Dekeyser, Anne Deschamps, Corinne Donzaud, Damien Dornic, Hasankiadeh Q. Dorosti, Doriane Drouhin, Thomas Eberl, Umberto Emanuele, Jean-Pierre Ernenwein, Stéphanie Escoffier, Paolo Fermani, Marcelino Ferri, Vincenzo Flaminio, Florian Folger, Ulf Fritsch, Jean-Luc Fuda, Salvatore Galatà, Pascal Gay, Giorgio Giacomelli, Valentina Giordano, Juan-Pablo Gómez-González, Kay Graf, Goulven Guillard, Garadeb Halladjian, Gregory Hallewell, Hans van Haren, Joris Hartman, Aart J. Heijboer, Yann Hello, Juan Jose Hernández-Rey, Bjoern Herold, Jurgen Hößl, Ching-Cheng Hsu, Marteen de Jong, Matthias Kadler, Oleg Kalekin, Alexander Kappes, Uli Katz, Oksana Kavatsyuk, Paul Kooijman, Claudio Kopper, Antoine Kouchner, Ingo Kreykenbohm, Vladimir Kulikovskiy, Robert Lahmann, Patrick Lamare, Giuseppina Larosa, Dario Lattuada, Gordon Lim, Domenico Lo Presti, Herbert Loehner, Sotiris Loucatos, Salvatore Mangano, Michel Marcelin, Annarita Margiotta, Juan Antonio Martinez-Mora, Athina Meli, Teresa Montaruli, Luciano Moscoso, Holger Motz, Max Neff, Emma nuel Nezri, Dimitris Palioselitis, Gabriela E. Păvălaş, Kevin Payet, Patrice Payre, Jelena Petrovic, Paolo Piattelli, Nicolas Picot-Clemente, Vlad Popa, Thierry Pradier, Eleonora Presani, Chantal Racca, Corey Reed, Giorgio Riccobene, Carsten Richardt, Roland Richter, Colas Rivière, Kathrin Roensch, Andrei Rostovtsev, Joaquin Ruiz-Rivas, Marius Rujoiu, Valerio G. Russo, Francisco Salesa, Augustin Sánchez-Losa, Piera Sapienza, Friederike Schöck, Jean-Pierre Schuller, Fabian Schussler, Rezo Shanidze, Francesco Simeone, Andreas Spies, Maurizio Spurio, Jos J. M. Steijger, Thierry Stolarczyk, Mauro G. F. Taiuti, Simona Toscano, Bertrand Vallage, Véronique Van Elewyck, Giulia Vannoni, Manuela Vecchi, Pascal Vernin, Guus Wijnker, Jorn Wilms, Els de Wolf, Harold Yepes, Dmitry Zaborov, Juan De Dios Zornoza, Juan Zúñiga

**Affiliations:** 1 Aix Marseille Université, CNRS/INSU, IRD, Mediterranean Institute of Oceanography (MIO), UM 110, Marseille, France; 2 Université de Toulon, CNRS/INSU, IRD, Mediterranean Institute of Oceanography (MIO), UM 110, La Garde, France; 3 GRC Geociències Marines, Departament d'Estratigrafia, Paleontologia i Geociències Marines, Facultat de Geologia, Universitat de Barcelona, Campus de Pedralbes, Barcelona, Spain; 4 Université de Perpignan, CNRS-INSU, CEFREM UMR5110, Perpignan, France; 5 Université Pierre & Marie Curie, CNRS-INSU, LOV UMR7093, Villefranche-sur-mer, France; 6 Université Pierre & Marie Curie, CNRS-INSU, Institut Pierre Simon Laplace, LOCEAN UMR 7159, Paris, France; 7 IFIC – Instituto de Física Corpuscular, Edificios Investigación de Paterna, CSIC – Universitat de València, Apdo. de Correos, Valencia, Spain; 8 CPPM – Aix-Marseille Université, CNRS/IN2P3, Marseille, France; 9 GRPHE – Institut universitaire de technologie de Colmar, Colmar, France; 10 Technical University of Catalonia, Laboratory of Applied Bioacoustics, Barcelona, Spain; 11 INFN – Sezione di Genova, Genova, Italy; 12 Friedrich-Alexander-Universität Erlangen-Nürnberg, Erlangen Centre for Astroparticle Physics, Erlangen, Germany; 13 Direction des Sciences de la Matière – Institut de recherche sur les lois fondamentales de l'Univers – Service d'Electronique des Détecteurs et d'Informatique, CEA Saclay, Gif-sur-Yvette, France; 14 Institut d'Investigació per a la Gestió Integrada de les Zones Costaneres (IGIC) – Universitat Politècnica de València, Gandia, Spain; 15 Nikhef, Amsterdam, The Netherlands; 16 APC, Université Paris Diderot, CNRS/IN2P3, CEA/IRFU, Observatoire de Paris, Sorbonne Paris Cité, Paris, France; 17 Aix Marseille Université, CNRS, LAM (Laboratoire d'Astrophysique de Marseille) UMR 7326, Marseille, France; 18 INFN – Sezione di Bologna, Bologna, Italy; 19 Dipartimento di Fisica dell'Università, Bologna, Italy; 20 INFN – Sezione di Pisa, Pisa, Italy; 21 INFN -Sezione di Roma, Roma, Italy; 22 Dipartimento di Fisica dell'Università La Sapienza, Roma, Italy; 23 Clermont Université, Université Blaise Pascal, CNRS/IN2P3, Laboratoire de Physique Corpusculaire, IN2P3-CNRS, Clermont-Ferrand, France; 24 INAF-IASF, Bologna, Italy; 25 Géoazur – Université de Nice Sophia-Antipolis, CNRS/INSU, IRD, Observatoire de la Côte d'Azur and Université Pierre et Marie Curie, Villefranche-sur-Mer, France; 26 INFN – Sezione di Bari, Bari, Italy; 27 INFN – Laboratori Nazionali del Sud (LNS), Catania, Italy; 28 Université Paris-Sud, Orsay, France; 29 Kernfysisch Versneller Instituut (KVI), University of Groningen, Groningen, The Netherlands; 30 Dipartimento di Fisica dell'Università Pisa, Italy; 31 Royal Netherlands Institute for Sea Research (NIOZ), Texel, The Netherlands; 32 Dr. Remeis Sternwarte & ECAP, Bamberg, Germany; 33 Universiteit Utrecht, Faculteit Betawetenschappen, Utrecht, The Netherlands; 34 Universiteit van Amsterdam, Instituut voor Hoge-Energie Fysika, Amsterdam, The Netherlands; 35 Moscow State University, Skobeltsyn Institute of Nuclear Physics, Moscow, Russia; 36 INFN – Sezione di Catania, Catania, Italy; 37 Dipartimento di Fisica ed Astronomia dell'Università, Catania, Italy; 38 Direction des Sciences de la Matière – Institut de recherche sur les lois fondamentales de l'Univers – Service de Physique des Particules, CEA Saclay, Gif-sur-Yvette, France; 39 University of Wisconsin – Madison, Madison, Wisconsin, United States of America; 40 Institute for Space Sciences, Bucharest, Romania; 41 IPHC-Institut Pluridisciplinaire Hubert Curien – Université de Strasbourg et CNRS/IN2P3, Strasbourg, France; 42 ITEP – Institute for Theoretical and Experimental Physics, Moscow, Russia; 43 Dipartimento di Fisica dell'Università, Genova, Italy; Heriot-Watt University, United Kingdom

## Abstract

The deep ocean is the largest and least known ecosystem on Earth. It hosts numerous pelagic organisms, most of which are able to emit light. Here we present a unique data set consisting of a 2.5-year long record of light emission by deep-sea pelagic organisms, measured from December 2007 to June 2010 at the ANTARES underwater neutrino telescope in the deep NW Mediterranean Sea, jointly with synchronous hydrological records. This is the longest continuous time-series of deep-sea bioluminescence ever recorded. Our record reveals several weeks long, seasonal bioluminescence blooms with light intensity up to two orders of magnitude higher than background values, which correlate to changes in the properties of deep waters. Such changes are triggered by the winter cooling and evaporation experienced by the upper ocean layer in the Gulf of Lion that leads to the formation and subsequent sinking of dense water through a process known as “open-sea convection”. It episodically renews the deep water of the study area and conveys fresh organic matter that fuels the deep ecosystems. Luminous bacteria most likely are the main contributors to the observed deep-sea bioluminescence blooms. Our observations demonstrate a consistent and rapid connection between deep open-sea convection and bathypelagic biological activity, as expressed by bioluminescence. In a setting where dense water formation events are likely to decline under global warming scenarios enhancing ocean stratification, *in situ* observatories become essential as environmental sentinels for the monitoring and understanding of deep-sea ecosystem shifts.

## Introduction

The deep-sea ecosystem is unique because of its permanent darkness, coldness, high pressure and scarcity of carbon and energy to sustain life. Most of its biological activity relies on the arrival of carbon in the form of organic matter from surface waters. Ninety percent of the numerous pelagic organisms that inhabit the deep ocean are capable of emitting light [1] through the chemical process of bioluminescence, which appears to be the most common form of communication in this remote realm [1], [2], [3]. Deep-sea bioluminescence is also viewed as an expression of abundance and adaptation of organisms to their environment [4]. Marine bioluminescent organisms include a variety of distinct taxa [4]. When stimulated mechanically or electrically, eukaryotic bioluminescent organisms emit erratic luminous flashes, and also spontaneous flashes to attract prey and mates for recognition of congeners or for defence purposes [1], [3], [4]. In contrast, luminescent bacteria are unaffected by mechanical stimulation and can glow continuously for many days under specific growth conditions [5], [6]. Bioluminescent bacteria occur in marine waters as free-living forms, symbionts in luminous organs of fishes and crustaceans and attached to marine snow aggregates sinking through the water column [5], [7]. During micro-algae blooms, strong bioluminescence produced by colonies of bacteria could even lead to spectacular marine phenomena such as “milky seas” in surface waters [6].

Bioluminescence sources have been observed and quantified over the last three decades using a variety of observational platforms and instruments such as manned submersibles [1] and autonomous underwater vehicles [8], *in situ* high sensitivity cameras [9], [10], underwater photometers [7], [11], [12], and remote satellite imagery [6]. In most cases, deep–sea bioluminescence is triggered and observed after external mechanical stimulation using, for instance, pumped flows through turbulence-generating grids [13] or downward moving grids that collide with the organisms [10]. While these procedures provide crucial information on the nature and distribution of deep-sea bioluminescent organisms in the water column [3 and references therein], they are not suited to investigate the temporal variability of naturally occurring light production (i.e. non artificially triggered) at specific sites over long periods of time, which requires sustained high frequency *in situ* measurements.

**Figure 1:**
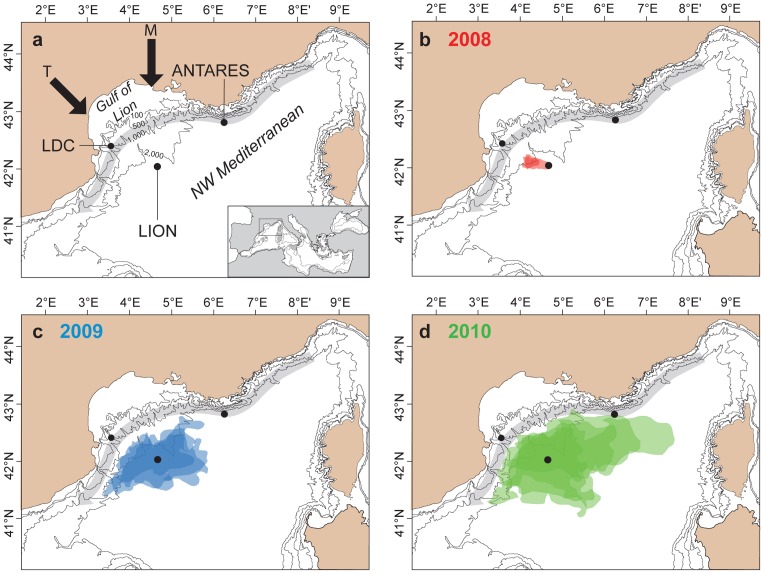
Map of the NW Mediterranean Sea showing the location of the ANTARES, LION and Lacaze-Duthiers Canyon (LDC) sites (a) as well as the extension of open-sea convection area in the Gulf of Lion and beyond from 2008 to 2010 (b–d). The boundaries of the convection area in winter 2008 (red in b), 2009 (blue in c) and 2010 (green in d) are derived from MODIS-Aqua satellite-based surface Chlorophyll-a concentration images. The limits of the convection area for each of the three successive winters correspond to their maximum extents during periods of deep water formation measured at the LION site (see Text S1 and Fig. S5). Black arrows indicate the direction of the two main continental winds leading to the cooling and subsequent sinking of surface waters: Mistral (M) and Tramontane (T). The grey arrow indicates the path of the cyclonic surface mesoscale Northern Current bordering the open-sea convection region.

An unanticipated application of underwater neutrino telescopes is to provide direct measurements of bioluminescence in the deep-sea [14], [15], [16]. A neutrino telescope aims at detecting the faint Cherenkov light emission radiated by elementary charged particles called muons that are produced by neutrino interactions. Darkness, transparency and water shielding against cosmic ray muons make the deep-sea an ideal setting for a neutrino telescope. Here we make use of both the high frequency bioluminescence and hydrological time-series of the cabled ANTARES neutrino telescope [17] located 40 km off the French coast (42°48′N, 6°10′E) at 2,475 in the NW Mediterranean Sea (Fig. 1a).

The NW Mediterranean Sea is one of the few regions in the world's ocean where both dense shelf water cascading and open-sea convection take place [18], [19], [20], [21] (Fig. 1a). This results in the formation of deep water owing to the combination of atmospheric forcing and regional circulation that lead the water column to overturn [19], [20], [22]. Dense deep water formation occurs during late winter and early spring due to cold, strong and persistent northern winds (Mistral and Tramontane) causing surface cooling of the Modified Atlantic Water (MAW) both on the shelf and over the deep basin. When the cooled shallow waters on the shelf become denser than the ambient waters, they start sinking, overflow the shelf edge, and cascade downslope until they reach their density equilibrium depth, which may vary from 150 m to more than 2,000 m [20], [23]. At the same time, convection in the adjacent deep basin involves a progressive deepening of the upper ocean mixed layer, which first reaches the warmer and saltier underlying Levantine Intermediate Water (LIW) and eventually extends all the way down to the basin floor, should the atmospheric forcing be intense enough [19]. Both processes and the subsequent renewal of the Western Mediterranean Deep Water (WMDW) show a high interannual variability because of their sensitivity to atmospheric conditions [18], [24]. The newly-formed deep water (nWMDW) resulting from both dense shelf water cascading and open-sea convection has been observed to spread over the deep basin floor within months [22], [24], [25], [26]. Studies about the response of deep ecosystems to such processes are scarce and focus on the impact of dense shelf water cascading on benthic and epi-benthic organisms [27], [28]. Other recent works highlight how deep water formation triggers the resuspension of deep-sea sediments, including organic matter [21], and the development and spreading of a thick bottom layer loaded with resuspended particulate matter across the NW Mediterranean Basin as a result of dense shelf water cascading [29].

Here we present compelling evidence of the quick response of the deep-sea pelagic ecosystem to seasonal atmospheric forcing leading to dense water formation and sinking, expressed by particularly intense bioluminescence events captured by neutrino telescope photomultiplier tubes. Observations on bioluminescence are supported by a two and a half years long unique and consistent record of hydrological and hydrodynamical variables obtained at the ANTARES deep-sea neutrino telescope itself but also at two independent mooring arrays equally located in the deep NW Mediterranean Sea.

## Results and Discussion

### Bioluminescence blooms at the ANTARES site

**Figure 2:**
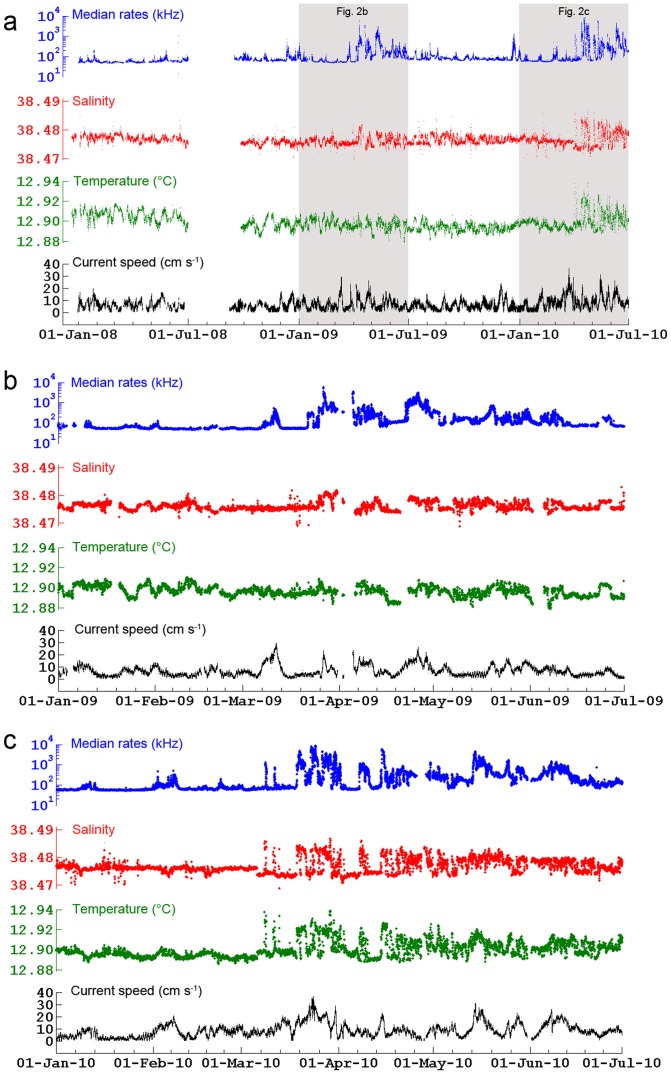
Time series measured at the ANTARES IL07 mooring line. **(a)** Median PMT counting rates (log scale), salinity, potential temperature and current speed from December 2007 to June 2010. Shading indicates periods (**b**) from January to June 2009 and (**c**) from January to June 2010, in which bioluminescence blooms were recorded. The lack of data from June 24 to September 6, 2008 is due to a cable technical failure.

We report time-series measurements of light intensity expressed in median counting rates on photomultiplier tubes as well as temperature, salinity and current speed from December 2007 to June 2010 (Fig. 2a), collected between 2,190 and 2,375 depth in the ANTARES IL07 mooring line (see Methods and Fig. S1). While the light intensity background rate is predominantly between 40 and 100 kHz, which mainly includes the ^40^K rate (see Methods and Fig. S2), two remarkable bioluminescence events reaching up to 9,000 were recorded between March and July in 2009 and 2010 (Fig. 2). Because of their high intensity and duration we call these events “bioluminescence blooms”, defined here as periods with PMT median rates higher than 600 kHz, i.e. higher than the 96^th^ percentile of the entire PMTs record.

Our records show that bioluminescence primarily increases with current speed, which is due to mechanical stimulation either by impacts of small-sized organisms and particles on the PMTs [16], [30] or by the reaction of organisms to enhanced turbulent motion in the wakes of the PMTs [14], [15], [16]. However, current speed alone fails to explain the complete record of bioluminescent activity since, for moderate current speeds, differences in the median rates of up to one order of magnitude are observed in 2009 (Fig. 2b) and 2010 (Fig. 2c). For instance, on March 8, March 11 and April 8–12, 2010, bioluminescence peaks at 800 to 1300 while current speeds are rather low, from 10 to 15 cm s^−1^ (Fig. 2c) a speed range usually associated to median rates of around 100. These bioluminescence bursts clearly correspond to significant increases in both potential temperature (Δθ = 0.03–0.05°C) and salinity (ΔS = 0.005–0.015). As the deep water mass at the ANTARES site is the WMDW, characterized in 2008 by a narrow range of temperature and salinity (θ = 12.89–12.92°C, S = 38.474–38.479), the increases above the normal range of variation observed in 2009 and 2010 are indicative of the intrusion of a distinct water mass (Fig. 2, see Text S1 and Fig. S3). It is noteworthy that neither deep-water thermohaline modification nor bioluminescence blooms were recorded in 2008 (Fig. 2a).

**Figure 3:**
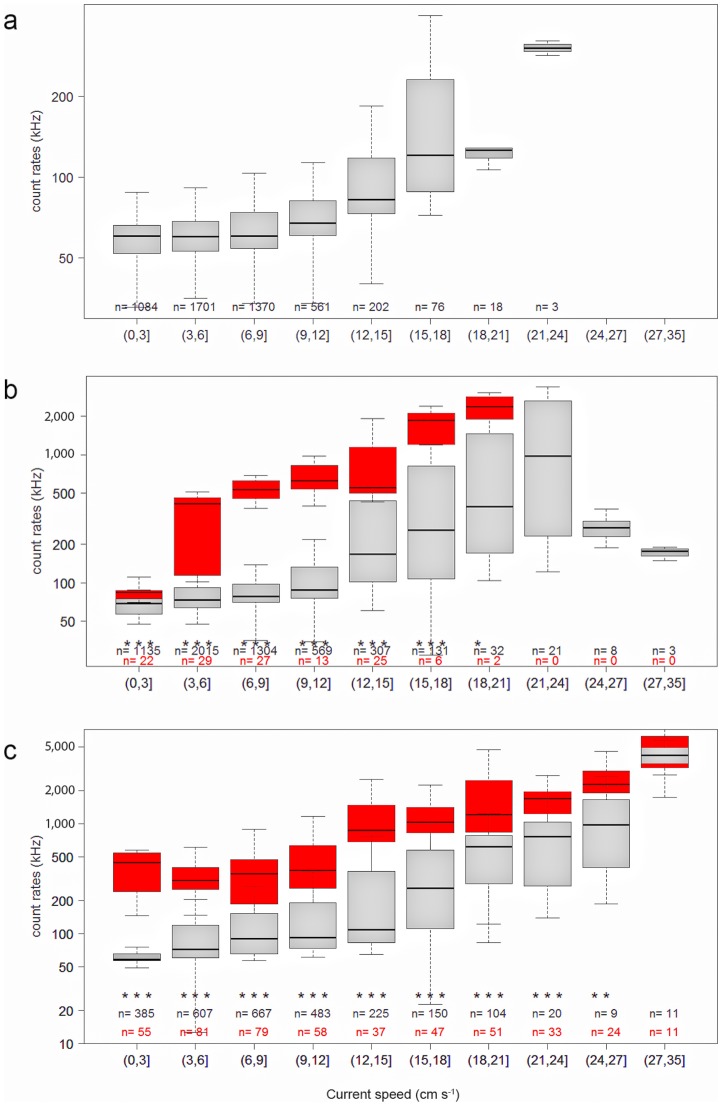
Links between bioluminescence, current speed and the modification of the properties of the Western Mediterranean Deep Water (WMDW). Box-and-whisker plot of median PMT counting rates (log scale) versus current speed classes for salinities higher (red) or lower (grey) than 38.479 for data recorded in (**a**) 2008, (**b**) 2009 and (**c**) between January and June 2010. The salinity threshold of 38.479 is used as a marker of the intrusion of newly formed deep water at the ANTARES site. While bioluminescence increases with current speed, it is also enhanced by the modification of WMDW (red box-plots). The top and bottom of each box-plot represent 75% (upper quartile) and 25% (lower quartile) of all values, respectively. The horizontal line is the median. The ends of the whiskers represent the 10^th^ and 90^th^ percentiles. Outliers are not represented. The statistical comparison between the two box-plots (red and grey) in each current class is given by the Kruskal-Wallis test: the observed difference between the two samples is significant beyond the 0.05 (*), the 0.01 (**) and the 0.001 (***) levels. The absence of an asterisk in some current classes indicates that the difference between the two box-plots is not significant. The number of measurements for salinity lower or higher than 38.479 is given in black or in red, respectively. Note the different scales of figures a, b and c.

To illustrate the link between the intrusion of newly formed deep water and high bioluminescence, we use a salinity threshold of 38.479 as marker of such intrusions at the ANTARES site. This value has been defined using a statistical decision tree (Fig. S4) and also corresponds to the 96^th^ percentile of the entire salinity record. Bioluminescence data, divided into two groups above and below this salinity threshold, are presented as box-and-whisker plots versus current speed classes (Fig. 3). Close examination of Figure 3 shows that bioluminescent activity is enhanced by both increasing current speed and the renewal of the deep water. Indeed, on the one hand, the bioluminescence rates increase with current speed for each of the two bioluminescence data groups (grey and red box-and-whisker plots) and on the other hand, bioluminescence rates are always higher for new deep water (red boxes, S>38.479) than for pre-existing deep water (grey boxes, S<38.479). The Kruskal-Wallis test performed on the box-and-whisker plots attests that the red and grey boxes are significantly different (p<0.001) for current speeds up to 18 and 24 cm s^−1^ in 2009 and 2010, respectively (Fig. 3b–3c), which means that bioluminescence rates are dependent on water mass properties too. This is illustrated, for instance, by the 2010 record (Fig. 3c), which shows that the median bioluminescence rate for the 0–3 cm s^−1^ current range is about 60 kHz for the existing deep water (grey box-plots), while it reaches 400 kHz within the new deep water (red box-plots). Bioluminescent bacteria, which are not affected by mechanical stimulation [5], [14] and are able to glow continuously under specific conditions [5], [6], are excellent candidates as main contributors to these bioluminescence blooms.

### Deep-water convection in the NW Mediterranean Sea

To determine the origin of the newly formed deep water observed at the ANTARES site in 2009 and 2010, we investigated whether dense shelf water cascading and/or open-sea convection occurred in winter months.

**Figure 4:**
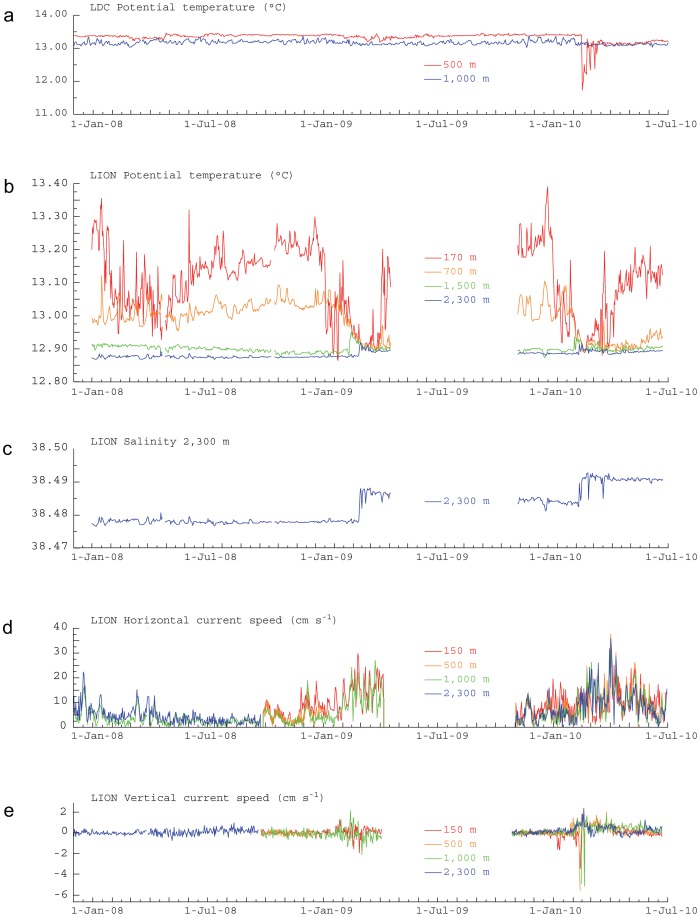
Time series of oceanographic parameters measured at the Lacaze-Duthiers Canyon (LDC) and the open-sea convection region in the Gulf of Lion (LION) from January 2008 to June 2010. (**a**) Potential temperature at 500 and 1,000 m depth at the LDC mooring site and (**b**) from various water depths at the LION site, jointly with (**c**) salinity at 2,300 m depth, (**d**) horizontal current speed and (**e**) vertical current speed from various water depths at the LION site. The four levels of temperature measurements at LION presented here are a sub-set of measurement depths (see Fig. S1). Essentially stable temperatures in the deepest layers in 2008 show that open-sea convection reached only 700 m and did not modify the deep water in the study area. In contrast, strong convection events, reaching 2,300 m depth, occurred during February-March 2009 and 2010 with an abrupt cooling of the upper water column and an increase in temperature and salinity in the deep layers. A concurrent increase in current speed was also noticed in winter 2009 and 2010. The 5-month long data gap in 2009 is due to a damaging of the mooring line during the April 2009 recovery, which induced a postponement of its redeployment to September 2009.

Instrumented mooring lines located at the center of the deep convection region (LION site at 42°02′N, 04°41′E; Fig. 1a) and in Lacaze-Duthiers Canyon (LDC site at 42°26′N, 03°33′E; Fig. 1a) provided temperature, salinity and current speed time-series from different water depths (Fig. 4) synchronous to the ANTARES record. While no deep (≥1,000 m) dense shelf water cascading took place during the study period (Fig. 4a), bottom-reaching open-sea convection was observed in the basin down to 2,300 m depth during wintertime in 2009 and 2010, which led to the homogenization of the water column (Fig. 4b). Increases in deep-water temperature (Fig. 4b) and salinity (Fig. 4c) are due to the mixture of sinking cold surface water with warmer and saltier LIW. In winter 2008, open-sea convection only affected the upper 1,000 m of the water column and did not alter the deep water mass. Current measurements showed the strong barotropic character of horizontal velocities (Fig. 4d) and high vertical velocities (Fig. 4e) during intense mixing periods. Once the surface forcing abates, convection ceases and intense sub-mesoscale eddies carry discrete volumes of the newly formed deep water away from the convection area [26]. The delay between the appearance of the thermohaline anomalies at the LION site in late winter and their arrival at the ANTARES site in spring is compatible with the spreading of the newly-formed deep water in the Gulf of Lion and subsequent mixing with pre-existing deep water [22], [24], [25], [26]. Further mixing could take place at the ANTARES site due to enhancement of vertical motion by the interaction of instabilities in the surface cyclonic Northern Current with the topography of the continental slope [31]. The area of open-sea convection, as obtained from satellite imagery (see Fig. S5), was much smaller during winter 2008 than in 2009 and 2010 when it covered most of the deep Gulf of Lion (Figs. 1b–d). Furthermore, it was larger and closer to the ANTARES site in 2010 than in 2009 (Fig. 1c–d), which may explain why the signature of new deep-water recorded at the ANTARES site is stronger in 2010 than in 2009 (Fig. 2).

### Link between bioluminescence blooms and deep-water convection

All evidence points to deep-water formation by open-sea convection in the Gulf of Lion as the cause of the renewal of deep water at the ANTARES site that triggered the bioluminescence blooms observed in 2009 and 2010.

During and in the aftermath of the convection period large amounts of organic matter, both in particulate (POC) and dissolved (DOC) form, are exported from the productive upper ocean layer down to the deep [21], [32], [33]. The resuspension of soft sediments covering the deep seafloor by bottom currents during the reported period could also inject organic matter into the deep-water mass [21], [32]. Changes in DOC concentration at the ANTARES site are shown by discrete measurements carried out at 2,000 m depth during oceanographic cruises from December 2009 to July 2010 (Fig. S6). DOC concentration significantly increased from 42±1 µM in December 2009, prior to the convection period, to 63±1 µM in March and May 2010 when the new deep water mass occupied the ANTARES site, concurrently with higher oxygen contents in bottom waters between March and mid-June 2010. Subsequently, DOC concentration decreased to 45 in mid-June and mid-July 2010 (Fig. S6). Such an injection of organic matter into the deep water mass has the potential to fuel the deep-sea biological activity, thus stimulating bioluminescence activity. The increase in DOC concentration matches with observations reported by Santinelli et al. [33] for different regions of the Mediterranean Sea where deep convection occurs. These authors showed a high mineralization rate of DOC in recently ventilated deep waters, which is mainly attributed to bacteria. Bioluminescent bacteria were isolated at the ANTARES site during a previous period of high bioluminescent activity in 2005 [34]. Amongst them, we identified a piezophilic strain, *Photobacterium phosphoreum* ANT-2200 [34], [35], *P. phosphoreum* being the dominant bioluminescent species in the Mediterranean Sea [36]. These luminous bacteria likely represent the main organisms responsible for the higher level of bioluminescence detected at the ANTARES site. Such contribution is especially noticed when the current speed is low within the convection season (Fig. 3b–3c). Finally, the flow associated with deep convection events might likely carries significant amounts of bioluminescent organisms too, which can also contribute to the bioluminescence blooms observed in 2009 and 2010 due to their collision with PMTs and/or their stimulation by turbulent motion in the wakes of PMTs when current speed is high.

## Conclusions

We present evidence for seasonal episodes of dense water formation driven by atmospheric forcing being a major vector in fuelling the deep-sea pelagic ecosystem and inducing bioluminescence blooms after a fast transfer of the ocean surface signal. Since dense water formation occurs in other ocean regions worldwide [19], we anticipate that an enhancement of the deep pelagic ecosystem activity similar to that observed in the NW Mediterranean Sea occurs there too, challenging our understanding of the carbon dynamics in the ocean.

Dense water formation is likely to be altered by the on-going global warming. Recent models [37], [38] based on the A2 IPCC scenario indicate a strong reduction in the convection intensity in the Mediterranean Sea for the end of the 21^st^ century, which will induce a massive reduction in organic matter supply and ventilation of the deep basin. Hence changes in the deep Mediterranean ecosystem more intense than those already observed in both the Eastern [39], [40] and the Western Mediterranean [27] basins are forecasted for the near future, a situation that could also occur but remain unnoticed in other sensitive areas of the world ocean. Our results illustrate the potentially far-reaching multidisciplinary scientific and societal benefits of the installation of cabled deep-sea observatories in critical ocean areas.

## Methods

The ANTARES neutrino telescope comprises a three-dimensional array of 885 Hamamatsu R7081-20 photomultiplier tubes (PMTs) distributed on 12 mooring lines [17], [41]. These PMTs are sensitive to the wavelength range of 400–700 nm, which matches the main bioluminescence emission spectrum (440–540 nm) as reported in Widder [4]. An extra mooring line (named IL07) equipped with RDI 300 kHz acoustic Doppler current profilers, a conductivity-temperature-depth (SBE 37 SMP CTD) probe and PMTs was added to monitor environmental variables (Fig. S1). All moorings are connected to a shore-station via an electro-optical cable that provides real-time data transmission [42]. A dedicated program of bioluminescence monitoring was implemented to measure the total number of single photons detected every 13 ms for each PMT. To consistently compare PMT counting rates (bioluminescence) with oceanographic data (temperature, salinity, current speeds) considering the acquisition interval of the later (15 minutes), we calculated the median rates as a mathematical estimator of PMT counting rates. The median was selected instead of the arithmetic mean because of its higher robustness and least disturbance by extreme values. Median rates were expressed in thousands of photons per second or kHz (see Text S1 and Fig. S2a). The main light contributions recorded by PMTs result from dark noise, from Cherenkov radiation induced by the beta decay of ^40^K in seawater and from bioluminescence. The dark noise is about 3±1 kHz and remains constant with time [41]. The Cherenkov radiation induced by the beta decay of ^40^K in seawater produces a background of about 37±3 kHz [43], found to be constant within the statistical errors over a period of a few years [44], [45]. Therefore, all light increases over this constant background (40±3 kHz) can only be due to bioluminescence. The records of light intensity at IL07 are representative of those collected by the whole array of ANTARES PMTs (see Text S1 and Fig. S2b).

Potential temperature, salinity, horizontal and vertical current speeds (Fig. S1) at the LION mooring line were measured with SBE 37 SMP CTD probes and Nortek Aquadopp Doppler current-meters regularly spaced between the subsurface (150 m,) and the seabed (2350 m). Potential temperatures and vertical velocities were corrected for the current-induced tilting and deepening of the line. Hourly potential temperatures at the LDC mooring line were measured with the temperature sensor of Nortek Aquadopp Doppler current meters at 500 and 1,000 m depth.

Proper calibrations of the CTD probes were performed using the pre- and post-deployment calibrations made by the manufacturer. The intercomparison of instruments complied with quality-control procedures.

## Supporting Information

Figure S1 **Configuration of the mooring lines from which the data presented in this study were obtained.** They include the cabled IL07 ANTARES as well as the autonomous LION and Lacaze-Duthiers Canyon (LDC) mooring lines. Location is shown in Fig. 1.
(JPG)

Figure S2 **(a) Raw counting rates from one photomultiplier (PMT) on the IL07 line (ANTARES site).** Counts are expressed in thousands of photons per second (kHz). The median rate is computed for each 15-minute data sample (red horizontal line). The dataset shown in the figure was recorded on March 28th, 2010 with a median rate of 68 kHz and a current speed of 13 cm s−1. **(b) Median rates from the IL07 PMT (red) and mean of all median rates of the 885 ANTARES PMTs (blue) from January to April 2009.**
(JPG)

Figure S3 **Potential temperature versus salinity diagram of near-bottom CTD time-series at the ANTARES site from the IL07 line (red dots) and CTD profiles (lines) collected close to the ANTARES site.** (**a**) May 2007 to January 2009; (**b**) January to December 2009; and (**c**) December 2009 to January 2011. The data shown are from depths in excess of 1,000 m. Dotted lines correspond to potential density anomaly isolines in kg m^−3^.
(JPG)

Figure S4 **Regression tree for predicting the intensity of bioluminescence using oceanographic variables (salinity, temperature, current speed) and time dependence from December 2007 to July 2010.** Regression trees are statistical models that sub-divide or partition a set of explanatory variables X (salinity, temperature, current speed) to predict a targeted response variable Y (bioluminescence rate). The tree is drawn using a binary recursive algorithm. It divides Y data into two non-empty groups either X <a or X> a. The split which maximizes the deviance (or distance) is chosen, the data set split and the process is repeated. This is done until the terminal nodes are too small or too few to be split, the last groups decision here are set up by the user in order to get less than 5 sub-groups. Each of the terminal nodes are the mean of the predicted value Y. Using this method, 3 nodes and 4 classes have been defined from the 3 variables predicting the average bioluminescence intensity. This classification improves the maximal deviance interclass and minimal deviance intraclass using sampled time-series. Class 1 (mean 121.1 kHz), 2 (mean 435.0 kHz) and 3 (mean 552.4 kHz) described low empirical bioluminescence intensity mainly due to low sea current speed (below 19.04 cm s^−1^). However class 3 and 4 are firstly described by high current speed intensity (>19.04 cm s^−1^) but as a second environmental condition, the temperature threshold of 12.922°C divide these two classes between high (mean 1393.0 kHz) and highest (mean 5108.0 kHz) bioluminescence intensity.
(JPG)

Figure S5 **Illustrative ocean colour satellite images used to outline the limits of winter open-sea convection areas in the Gulf of Lion.** (**a**) Images plotted with a classical, full range, linear palette. (**b**) Images plotted with a simplified four level palette. The images shown correspond to days 1, 2, 7 and 18 February 2010, which are also transferred into Fig. 1b–d. White pixels are indicative of lack of data due to cloud coverage.
(JPG)

Figure S6 **Dissolved organic carbon and oxygen concentrations at the ANTARES site in 2010.** Dissolved Organic Carbon (DOC) was measured by high temperature combustion on a Shimadzu TOC 5000 analyzer [46]. A four point-calibration curve was performed daily with standards prepared by diluting a stock solution of potassium hydrogen phthalate in Milli-Q water. Procedural blanks run with acidified and sparged Milli-Q water ranged from 1 to 2 µM C and were subtracted from the values presented here. Deep seawater reference samples (provided by D. Hansell; Univ. Miami) were run daily (43.5 µM C, n = 4) to check the accuracy of the DOC analysis. Oxygen concentration time-series was obtained using an oxygen optode Anderaa® fitted on the IL07.
(TIF)

Text S1 Supplemental text information.
(DOCX)
