# Supplementary figures and images for: Deep-Sea Bioluminescence Blooms after Dense Water Formation at the Ocean Surface
Source: PLoS One. 2013 Jul 10;8(7):e67523. doi: 10.1371/journal.pone.0067523 (PMC3707865; doi:10.1371/journal.pone.0067523)

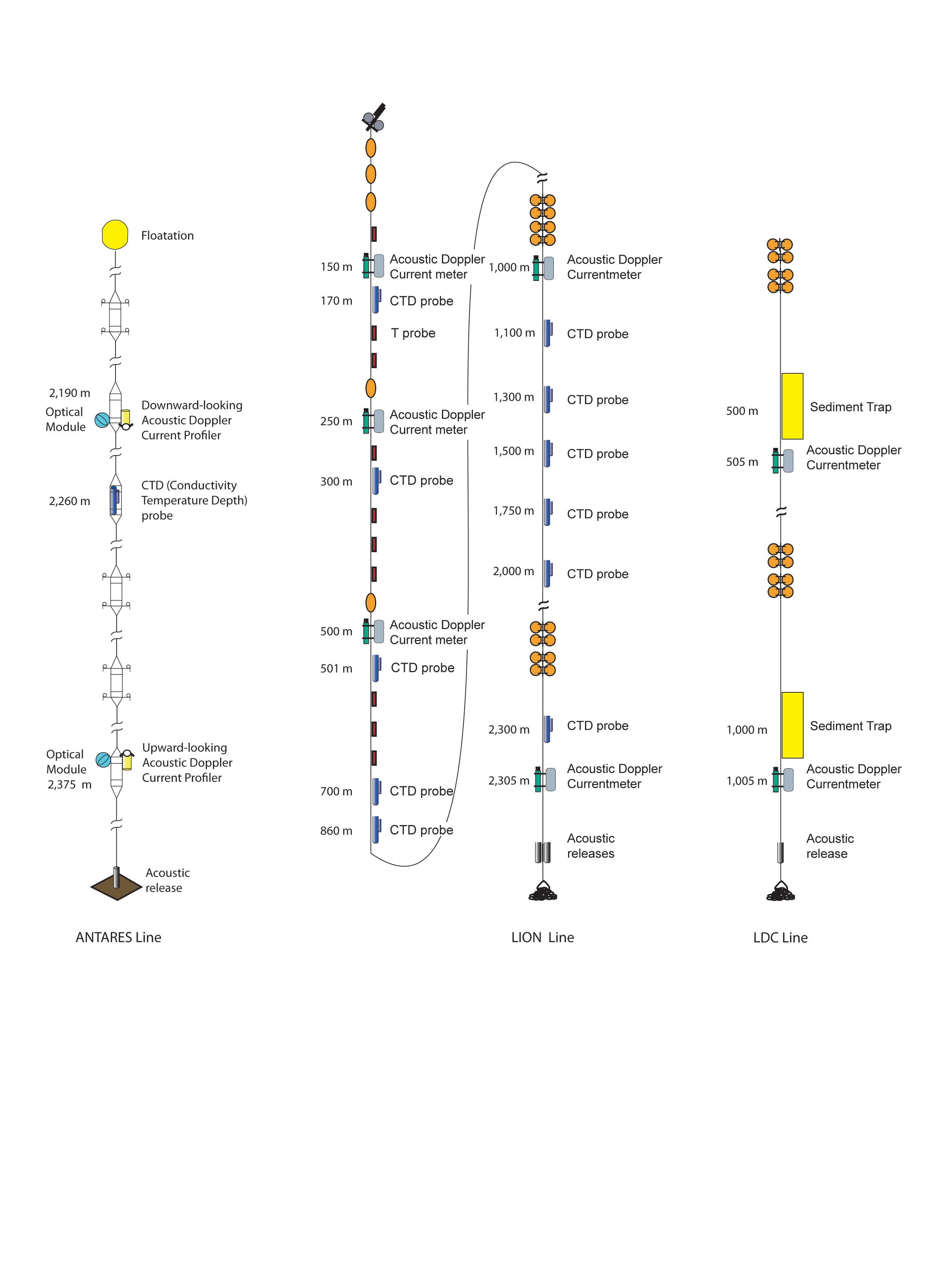

Supplement: Figure S1 — Configuration of the mooring lines from which the data presented in this study were obtained. They include the cabled IL07 ANTARES as well as the autonomous LION and Lacaze-Duthiers Canyon (LDC) mooring lines. Location is shown in Fig. 1. (JPG) [file pone.0067523.s001.jpg]

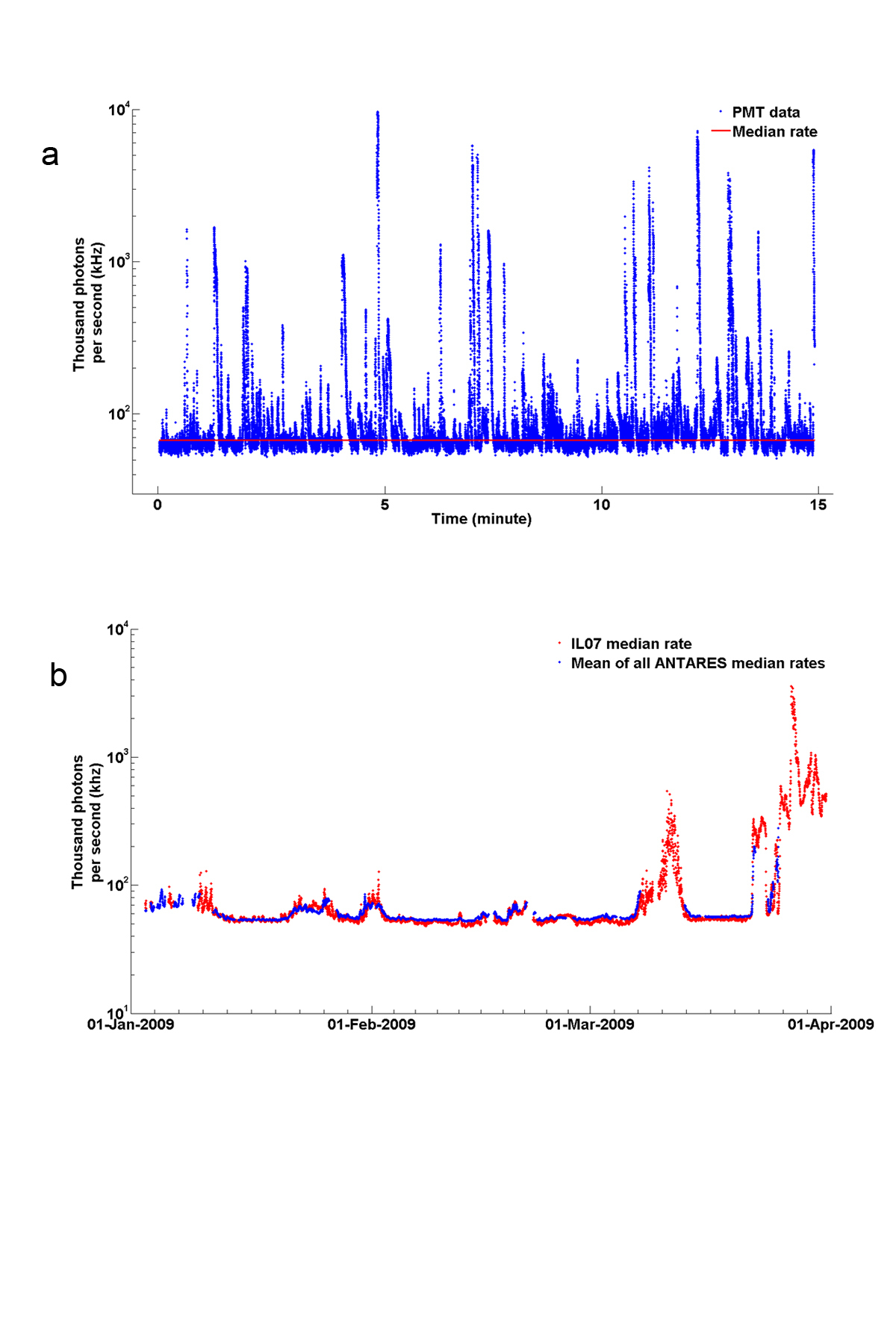

Supplement: Figure S2 — (a) Raw counting rates from one photomultiplier (PMT) on the IL07 line (ANTARES site). Counts are expressed in thousands of photons per second (kHz). The median rate is computed for each 15-minute data sample (red horizontal line). The dataset shown in the figure was recorded on March 28th, 2010 with a median rate of 68 kHz and a current speed of 13 cm s−1. (b) Median rates from the IL07 PMT (red) and mean of all median rates of the 885 ANTARES PMTs (blue) from January to April 2009. (JPG) [file pone.0067523.s002.jpg]

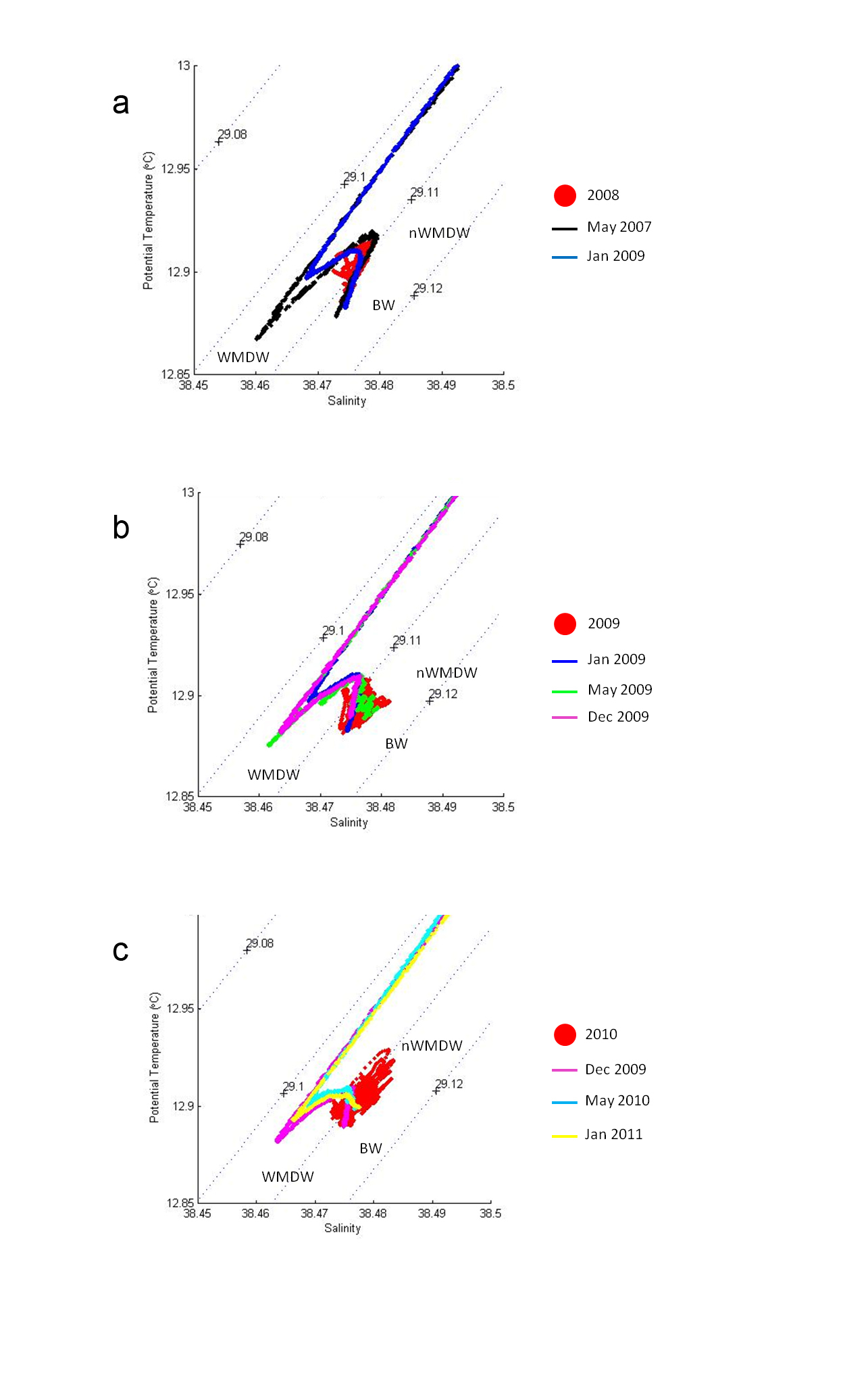

Supplement: Figure S3 — Potential temperature versus salinity diagram of near-bottom CTD time-series at the ANTARES site from the IL07 line (red dots) and CTD profiles (lines) collected close to the ANTARES site. (a) May 2007 to January 2009; (b) January to December 2009; and (c) December 2009 to January 2011. The data shown are from depths in excess of 1,000 m. Dotted lines correspond to potential density anomaly isolines in kg m−3. (JPG) [file pone.0067523.s003.jpg]

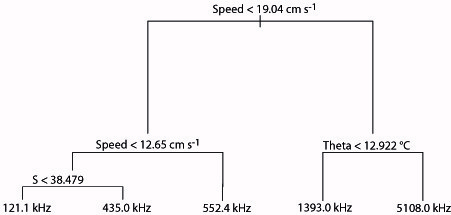

Supplement: Figure S4 — Regression tree for predicting the intensity of bioluminescence using oceanographic variables (salinity, temperature, current speed) and time dependence from December 2007 to July 2010. Regression trees are statistical models that sub-divide or partition a set of explanatory variables X (salinity, temperature, current speed) to predict a targeted response variable Y (bioluminescence rate). The tree is drawn using a binary recursive algorithm. It divides Y data into two non-empty groups either X a. The split which maximizes the deviance (or distance) is chosen, the data set split and the process is repeated. This is done until the terminal nodes are too small or too few to be split, the last groups decision here are set up by the user in order to get less than 5 sub-groups. Each of the terminal nodes are the mean of the predicted value Y. Using this method, 3 nodes and 4 classes have been defined from the 3 variables predicting the average bioluminescence intensity. This classification improves the maximal deviance interclass and minimal deviance intraclass using sampled time-series. Class 1 (mean 121.1 kHz), 2 (mean 435.0 kHz) and 3 (mean 552.4 kHz) described low empirical bioluminescence intensity mainly due to low sea current speed (below 19.04 cm s−1). However class 3 and 4 are firstly described by high current speed intensity (>19.04 cm s−1) but as a second environmental condition, the temperature threshold of 12.922°C divide these two classes between high (mean 1393.0 kHz) and highest (mean 5108.0 kHz) bioluminescence intensity. (JPG) [file pone.0067523.s004.jpg]

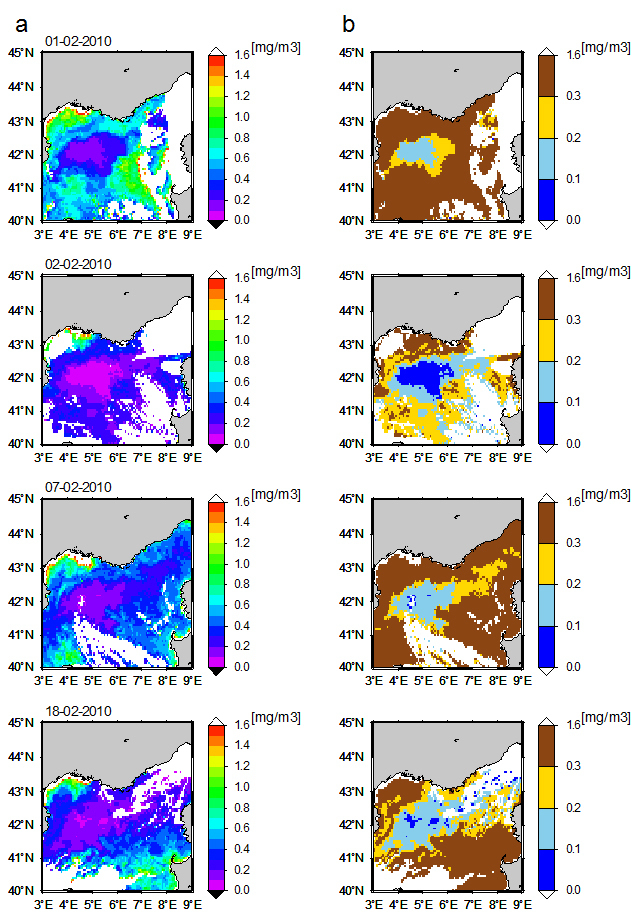

Supplement: Figure S5 — Illustrative ocean colour satellite images used to outline the limits of winter open-sea convection areas in the Gulf of Lion. (a) Images plotted with a classical, full range, linear palette. (b) Images plotted with a simplified four level palette. The images shown correspond to days 1, 2, 7 and 18 February 2010, which are also transferred into Fig. 1b–d. White pixels are indicative of lack of data due to cloud coverage. (JPG) [file pone.0067523.s005.jpg]

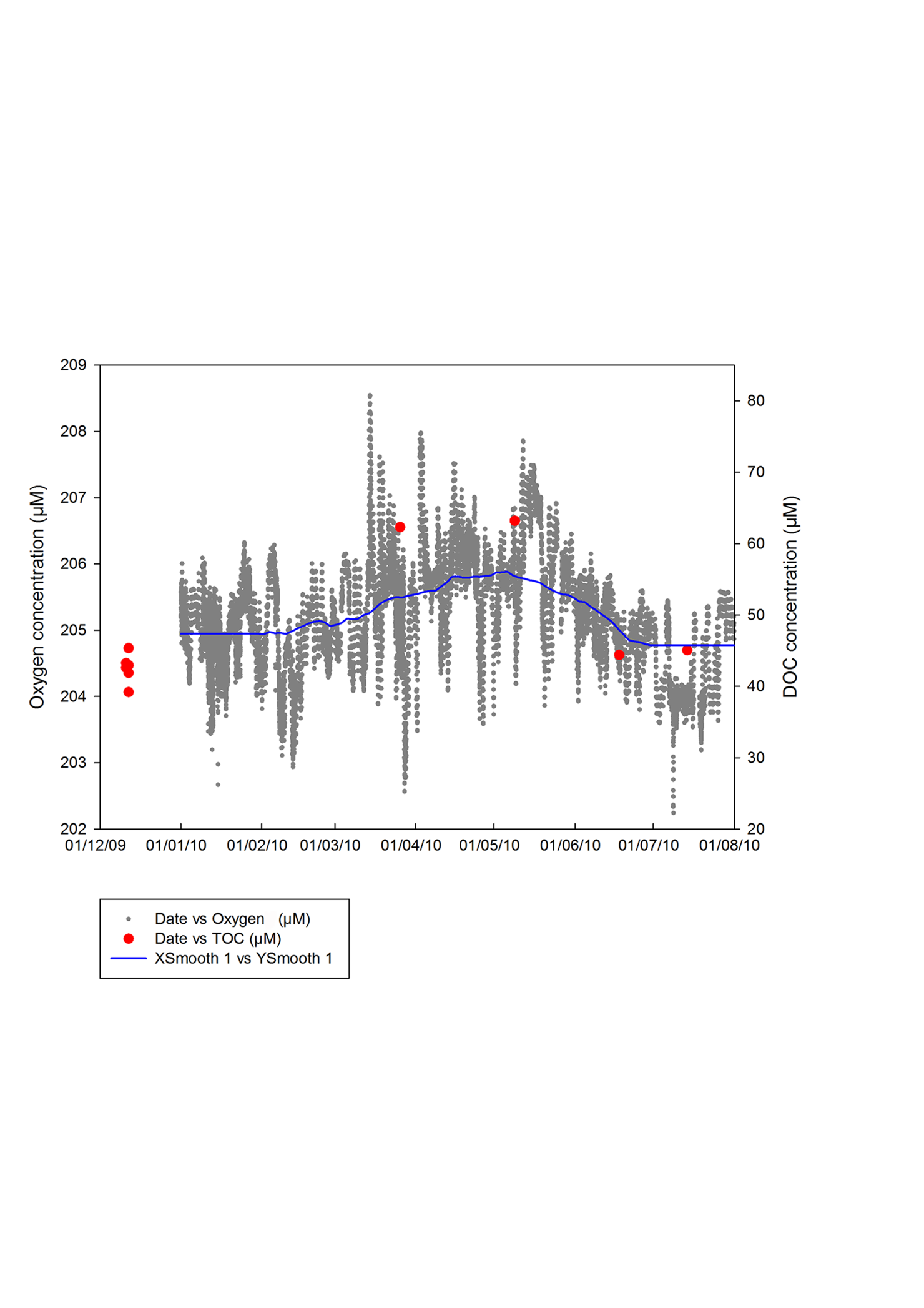

Supplement: Figure S6 — Dissolved organic carbon and oxygen concentrations at the ANTARES site in 2010. Dissolved Organic Carbon (DOC) was measured by high temperature combustion on a Shimadzu TOC 5000 analyzer [46]. A four point-calibration curve was performed daily with standards prepared by diluting a stock solution of potassium hydrogen phthalate in Milli-Q water. Procedural blanks run with acidified and sparged Milli-Q water ranged from 1 to 2 µM C and were subtracted from the values presented here. Deep seawater reference samples (provided by D. Hansell; Univ. Miami) were run daily (43.5 µM C, n = 4) to check the accuracy of the DOC analysis. Oxygen concentration time-series was obtained using an oxygen optode Anderaa® fitted on the IL07. (TIF) [file pone.0067523.s006.tif]
