# Supplementary material for: Deep-Sea Bioluminescence Blooms after Dense Water Formation at the Ocean Surface
Source: PLoS One. 2013 Jul 10;8(7):e67523. doi: 10.1371/journal.pone.0067523 (PMC3707865; doi:10.1371/journal.pone.0067523)
Supplement: Text S1 — Supplemental text information. (DOCX) [file pone.0067523.s007.docx]

**Text S1:** Supplemental text information

# Estimating photomultiplier tube (PMT) median rates

# Each ANTARES optical module housing a photomultiplier tube (PMT) records the total number of photons every 13 ms (Fig. S2a). Light intensity is expressed in thousands of photons per second (kHz). Its lowest value is an essentially constant contribution that comes mainly from Cherenkov radiation induced by the beta decay of ^40^K [1] (see Materials and methods). Light intensity variation from this background is entirely due to bioluminescence, including both spontaneous and stimulated bioluminescence bursts and bacterial glow. In the current study, only bioluminescence variations using the median rate computed over 15-minute periods have been considered. The median rate is defined as the value separating the higher half of the sample from the lower half, and is utilized to reduce the influence of outliers.

# PMTs in the IL07 mooring line considered for this work acquired data continuously, while the other 885 ANTARES PMTs dedicated to neutrino detection were turned off during periods of high bioluminescence. Consistency checking has shown that median rates computed from IL07 PMTs are representative of all ANTARES PMTs (Fig. S2b).

# Potential temperature - Salinity diagrams from the ANTARES site below 1,000m

# The hydrological properties of the water column were measured during several oceanographic cruises at the ANTARES site and were subsequently compared to the ANTARES time-series data to provide a water column frame for Eulerian (i.e. fixed-site) observations. The CTD-based potential temperature against salinity plots between May 2007 (black line) and January 2009 (blue line) (Fig. S3a) show three deep water masses: 1) the old Western Mediterranean Deep Water (WMDW), below 1,000m, within a range of salinity between 38.460 and 38.470 and a potential temperature range between 12.86 and 12.91°C; 2) a new warmer and saltier WMDW (nWMDW) between 1,700 and 2,100 m, with a salinity range centred at 38.475 ± 0.004 and a potential temperature ranging between 12.91 and 12.92°C; and 3) a colder and less saline bottom water (BW) below 2,100m, with a range of salinity centred at 38.473 ± 0.002 and a potential temperature ranging between 12.87 and 12.88°C. The nWMDW and BW water masses first observed in 2007 appeared after the intense winter convection and cascading events of 2005 and 2006 [2,3,4] (Fig. S3a). IL07 CTD time-series in 2008 (red dots, Fig. S3a) show weak salinity variations that correspond to the mixing of nWMDW and BW.

# CTD casts and near-bottom time-series obtained during 2009 (Fig. S3b) and 2010 (Fig. S3c) show a significant change in BW properties. CTD time-series show that the thermohaline properties of the BW fluctuated significantly, with a slight increase of salinity in 2009 (Fig. S3b) and strong increases in both salinity and temperature in 2010 (Fig. S3c). The CTD casts clearly show the arrival of warmer and saltier BW in May 2009 (Fig. S3b) and May 2010 (Fig. S3c) after winter offshore deep water formation in the nearby Gulf of Lion. The alteration of BW persisted in January 2011.

**Interannual variability of the convection area in the Gulf of Lion from satellite imagery**

Convection areas where deep water forms display a distinct signature on ocean colour satellite images [5,6]. The intense mixing associated with deep water formation events is accompanied by very low chlorophyll concentrations (<0.1 mg m^-3^) at the sea surface that are detectable by remote ocean colour sensors. One limitation of satellite images is that the actual depth of mixing cannot be inferred directly; i.e. they would give similar results in terms of chlorophyll concentrations for both intermediate (down to 500-600m) and deep (down to >2,000m) convection events. Thus remote and *in situ* observations such as those obtained by research cruises and mooring arrays must be combined. Cloud coverage may also prevent a fine time and space characterization of the dense water formation process involving continuous observation of the area of interest. The short duration (i.e. 1-2 weeks) of deep water formation events may prevent them being observed from space if cloudiness persists.

Estimates of the size of deep-water formation areas are, therefore, more trustworthy if they are obtained from cloud-free images. The instantaneous horizontal dimension of satellite images provides a reference frame for in situ data, which cannot be achieved from in situ observations alone. Satellite images also provide estimates of the interannual variability of deep-water formation processes in terms of areal extent and intensity.

Sea-surface chlorophyll-a concentration images (NASA 558 MODIS-Aqua satellite) for the January 2008-March 2010 period with more than 75% of cloud-free pixels have been used to outline the limits of the convection area for each of the three successive winters. The chlorophyll concentration scale ranges from 0 to 1.6 mg m^-3^ in Fig. S5a, where colours correspond to 0.1 mg m^-3^ intervals. White pixels are indicative of cloud coverage. Pixels with low chlorophyll concentrations, i.e. less than 0.2 mg m^-3^, have been selected as indicative of deep water formation, which is represented by light blue and dark blue colours in the four level colour palette of Fig. S5b. The 0.2 mg m^-3^ threshold was selected after close inspection of the images corresponding to the deep water formation events measured at the LION mooring line. Boundaries of the deep water formation area corresponding to specific days within each winter are plotted in Fig. 1 (6 to 9 February in 2008 (red), 25 and 28 January, and 4 and 13 February in 2009 (blue), and 1, 2, 7 and 18 February in 2010 (grey)).

**References**

1. Amram P, Anvar S, Aslanides E, Aubert JJ, Azoulay R, et al. (2000) Background light in potential sites for the ANTARES undersea neutrino telescope. Astropart Phys 13: 127-136.

2. Smith RO, Bryden HL, Stansfield K (2008) Observations of new western Mediterranean deep water formation using Argo floats 2004-2006. Ocean Science 4: 133-149.

3. Schroeder K, Gasparini GP, Tangherlini M, Astraldi M (2006) Deep and intermediate water in the western Mediterranean under the influence of the Eastern Mediterranean Transient. Geophys Res Lett 33: L21607.

4. Canals M, Puig P, Durrieu de Madron X, Heussner S, Palanques A, et al. (2006) Flushing submarine canyons. Nature 444: 354-357.

5. Morel A, André JM (1991) Pigment distribution and primary production in the western Mediterranean as derived from CZCS observations. J Geophys Res 96: 12685-12691.

6. Santoleri R, Banzon V, Marullo S, Napolitano E, D'Ortenzio F, et al. (2003) Year-to-year variability of the phytoplankton bloom in the southern Adriatic Sea (1998-2000): Sea-viewing Wide Field-of-view Sensor observations and modeling study. J Geophys Res 108: 8122.
